# A model-based information sharing protocol for profile Hidden Markov Models used for HIV-1 recombination detection

**DOI:** 10.1186/1471-2105-15-205

**Published:** 2014-06-19

**Authors:** Ingo Bulla, Anne-Kathrin Schultz, Christophe Chesneau, Tanya Mark, Florin Serea

**Affiliations:** 1 Institut für Mathematik und Informatik, Universität Greifswald, Walther-Rathenau-Straße 47, 17487 Greifswald, Germany; 2 Theoretical Biology and Biophysics, Group T-6, Los Alamos National Laboratory, Los Alamos, New Mexico, USA; 3 Institute of Microbiology and Genetics, University of Göttingen, Goldschmidtstr. 1, 37077 Göttingen, Germany; 4 Université de Caen, LMNO, CNRS UMR 6139, Bd Maréchal Juin, BP 5186, 14032 Caen Cedex, France; 5 University of Guelph, 50 Stone Road, Guelph, Ontario N1G 2W1, Canada; 6 Technical University Gheorghe Asachi, Faculty of Electrical Engineering, Power Engineering and Applied Informatics, Bld. Dimitrie Mangeron 23, 700050 Iasi, Romania

## Abstract

**Background:**

In many applications, a family of nucleotide or protein sequences classified into several subfamilies has to be modeled. Profile Hidden Markov Models (pHMMs) are widely used for this task, modeling each subfamily separately by one pHMM. However, a major drawback of this approach is the difficulty of dealing with subfamilies composed of very few sequences. One of the most crucial bioinformatical tasks affected by the problem of small-size subfamilies is the subtyping of human immunodeficiency virus type 1 (HIV-1) sequences, i.e., HIV-1 subtypes for which only a small number of sequences is known.

**Results:**

To deal with small samples for particular subfamilies of HIV-1, we introduce a novel model-based information sharing protocol. It estimates the emission probabilities of the pHMM modeling a particular subfamily not only based on the nucleotide frequencies of the respective subfamily but also incorporating the nucleotide frequencies of all available subfamilies. To this end, the underlying probabilistic model mimics the pattern of commonality and variation between the subtypes with regards to the biological characteristics of HI viruses. In order to implement the proposed protocol, we make use of an existing HMM architecture and its associated inference engine.

**Conclusions:**

We apply the modified algorithm to classify HIV-1 sequence data in the form of partial HIV-1 sequences and semi-artificial recombinants. Thereby, we demonstrate that the performance of pHMMs can be significantly improved by the proposed technique. Moreover, we show that our algorithm performs significantly better than Simplot and Bootscanning.

## Background

### Information sharing protocol

Profile Hidden Markov Models (pHMMs) are widely employed to model nucleotide or protein sequence families, in particular for database search [1,2]. In numerous applications (see e.g. [3-5]), a sequence family classified into subfamilies is given, with each subfamily being modeled separately by one pHMM (i.e. only the information comprehended in the respective subfamily is used). If enough information is available about each subfamily (i.e. it consists of a sufficient number of sequences) this approach is feasible. Otherwise, the model is unable to detect sequences stemming from subfamilies composed of too few sequences [4].

To overcome this drawback, one can use an information sharing protocol which makes use of the information available about other subfamilies to model subfamilies composed of only a few sequences. Although they are usually named differently, information sharing protocols are a widely used concept in bioinformatics and other fields. For example, deducing pseudocounts [6] or Dirichlet mixtures [7] from a complete multiple sequence alignment to model a prior distribution for position-wise nucleotide or protein emission probabilities, constitutes a protocol for sharing information between different columns of the multiple sequence alignment (MSA). In speech recognition, using the same parameters for different HMMs within complex systems of HMMs in order to reduce the complexity of the system is called parameter tying [8]. In fact, if a prior distribution in a Bayesian framework [9] is deduced from a superordinate entity, one can regard the estimation of probabilities characterizing a subordinate part of this entity by application of the estimated prior distribution as an information sharing protocol.

Sjölander et al. [3] suggested such a protocol for automated protein subfamily identification and classification. Their approach to model the emission frequencies of a protein subfamily *S* is based on the idea to add the nucleotide frequencies of other subfamilies *T* (in weighted form) to the ones of *S* as done with pseudocounts. Hereby, the added frequencies of *T* are weighted by the probability of the amino acid frequencies of *T* under a model whose emission probabilities are constructed based only on the frequencies of *S*.

### HIV classification

HIV-1 is classified into the four main phylogenetic groups M, N, O, and P. All four were introduced into humans by separate zoonotic events, whereby the sources were different simian immunodeficiency viruses from chimpanzees [10] and gorilla [11], respectively. The M group is the most intensively studied one as it is responsible for the HIV pandemic. This group is further divided into ten subtypes, whereby Subtype A and F are further subdivided into sub-subtypes [12]. Inter-subtype recombination is very frequent among HIV-1 subtypes [13]: Up to now, 55 circulating recombinant forms (CRFs) have been reported [14], i.e., recombinant forms exhibiting at least three epidemiological independent sequences.

The subtypes of HIV-1 Group M can be divided into two groups: On the one hand, the ones for which at least 30 full-length sequences are available (subtypes A–G), on the other hand, subtypes H, J, and K, for which up to now only four (subtypes H and J) or two (Subtype K), respectively, complete-length sequences have been sampled. Thus, we refer to the first group – subtypes A–G – as “large-size” subtypes and to the second one – subtypes H–K – as “small-size” subtypes in what follows. Moreover, 11 full-length and about 1000 partial sequences have been labeled as “unknown”. That is, these sequences do not form an independent subtype or CRF. One can expect that by further sampling activities new small-size subtypes will be found, by finding sequences which are closely related to sequences of an unknown subtype already known, as well as by discovering sets of epidemiological independent sequences which are unrelated to the sequences now available [11].

### jpHMM

Up to now, roughly 50 tools have been developed for the purpose of recognition of recombinants and breakpoint detection in HIV-1 sequences. Examples are Bootscanning [15], Simplot [16], Recco [17], the REGA HIV-1 Subtyping Tool [18], and MaxChi2 [19]. In 2006, Schultz et al. developed the jumping profile Hidden Markov Model (jpHMM), an algorithm for subtyping, recombination analysis, and breakpoint detection, which we applied to HIV-1 and hepatitis C virus sequences [4]. The jpHMM is composed of one pHMM for each viral subtype. In addition to the usual transitions within these pHMMs, it allows for so-called jumps between different pHMMs at nearby positions. Thus, it is possible to jump between states associated with different subtypes, depending on the local similarity of the query sequence to the subtypes. Although performing well for most input sequences, it did not correctly predict the HIV-1 subtypes of smaller size H, J, and K. Further analysis revealed that this deficit was due to the lack of an appropriate inter-subtype information sharing protocol, i.e., no information from the large-size subtypes was used to model the small-size subtypes.

### Content

The aim of this paper is to develop an information sharing protocol suitable for the application to HIV-1 Group M sequences. The introduced protocol is incorporated into the HIV recombination detection tool jumping profile Hidden Markov Model [4,20,21], short jpHMM. We investigate the influence of this modification on the performance of jpHMM. This application is of particular interest as reliable and accurate classification of HIV-1 (and other viral sequence data) facilitates to understand the influence of genetic diversity on host immune response, is crucial for epidemiological studies, and provides therapeutic decision support [22-24].

## Methods

### Small-size HIV subtypes

The effect of modeling subfamilies with very few sequences separately is illustrated in Table 1. It depicts this problem for the emission probabilities of a position in an alignment of HIV-1 sequences: Constructing a pHMM for each subtype separately, the emission probability of adenine for Subtype K is nearly 7% lower than the one for Subtype A.

**Table 1:** The effects of a small subtype size

**Subtype**	**Nucleotide**	**Frequency**	**Probability**
	A	89	99.83%
A	C	0	0.06%
	G	0	0.06%
	T	0	0.06%
	A	392	99.71%
C	C	1	0.27%
	G	0	0.01%
	T	0	0.01%
	A	2	93.18%
K	C	0	2.27%
	G	0	2.27%
	T	0	2.27%

Nucleotide frequencies and the derived emission probabilities for one column in an alignment of HIV-1 Group M sequences without using an information sharing protocol. The emission probabilities are estimated from the nucleotide frequencies using the pseudocounts $\vec{\alpha }=(\text{0.05, 0.05, 0.05, 0.05})$. We here restrict ourselves to 3 subtypes, A, C, and K.

Since a vast majority of columns in the HIV-1 MSA are strongly conserved over subtypes, it is very probable that in fact the most realistic way to model the emission probabilities in this example consists of using a joint emission probability for all subtypes. Hence, the modeled emission probabilities of Subtype K should be much closer to the ones of Subtype A and C. They should still be smaller (but to a lesser extent) since one has to account for the following (rather improbable) scenario: The nucleotide frequencies of Subtype K only coincidentally suggests that all subtypes should be modeled jointly, but the real emission probabilities are, in fact, considerably different.

### Existing approaches

A workaround for this kind of situation consists of using the same emission probabilities for all subtypes in case a column is completely conserved (notice that in our example this does not apply to the column under consideration). But given that there are about 2000 non-recombinant full-length HIV-1 Group M genomes reported by now, almost all columns in an alignment composed of all these sequences are not 100% conserved.

In a preparatory step, Sjölander’s approach performs a sequence weighting based on the total numbers of independent counts. Unfortunately, this procedure results in sequence weights that are far too low if applied to HIV nucleotide sequence data instead of protein amino acid data. Trying to circumvent this problem by leaving this step out causes the influence of other subtypes to become too strong. Since Sjölander’s approach is, in addition, an ad hoc one and aims at protein sequence data which are considerably more variable than HIV-1 sequences, we developed an alternative algorithm. This new algorithm is suitable for HIV-1 nucleotide sequence data and is derived from a probabilistic model of the interdependence of different HIV-1 subtypes.

### Biological motivation

Our model of the emission probabilities is based on the observation that for almost all sites in an HIV-1 MSA at least some of the subtypes share the same emission probabilities. In fact, for the majority of sites, it would be most plausible to assign equal emission probabilities to all subtypes. Neglecting the trivial case of all subtypes having the same emission probability assigned to them, the phenomenon that some but not all of the subtypes exhibit equal emission probabilities could be explained biologically as follows: Assume a site allows for more than one nucleotide to be present (i.e. at least two alleles are observed). Then, there are only very few characteristics of the virus which determine which degree of fitness the virus has for the different nucleotides possible at the respective site. Moreover, these characteristics take only very few values. As the characteristics and their values at a particular site are small in number, the number of different nucleotide distributions observed at the respective site is also small.

To clarify this reasoning, let us assume that for a site *i* in the MSA the dependence of the virus fitness on the nucleotide at site *i* is determined by a binary characteristics (values 0 and 1) of the virus. For example, it could be the case that some part of the virus can assume two distinct shapes, influencing which nucleotides can be present at site *i* such that the virus is able to survive. Then, the following might hold for the nucleotide distributions of the subtypes: 

• If the characteristics takes the value 0, it might be that the virus can only survive if adenine is present at site *i*. This leads to a nucleotide distribution where adenine has a probability very near to one.

• In case it takes the value 1 the virus might also be able to survive if cytosine is present, but with a significant disadvantage with respect to its fitness. This might lead to a nucleotide distribution where adenine has a probability of, say, about 90% and the one of cytosine is about 10%.

In this case we would observe two different nucleotide distributions at site *i*: One part of the subtypes will show one nucleotide distribution and the other subtypes will show another. In the following, we will call the different nucleotide distributions (resp. emission probabilities) at the site “source”. That is, in the example just given there are two sources.

### Emission probabilities

**Figure 1:**
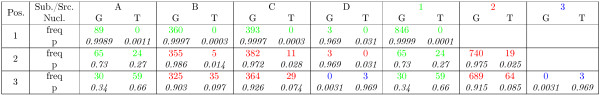
**Examples of the calculation of the emission probabilities.** Simplified example of position- and subtype-wise nucleotide frequencies of HIV and the emission probabilities derived from them using the presented information sharing protocol. For three sites the subtype-wise nucleotide frequencies for the four subtypes A-D are given on the left side of the table. Below them, the emission probabilities estimated based only on the frequencies of the respective subtype are shown, using pseudocounts $\vec{\alpha }=(0.1,0.1)$. The colors indicate which subtypes should be jointly modeled in order to get the most likely source combination. The nucleotide frequencies of the sources (i.e. the aggregated frequencies of the subtypes belonging to it) as well as the emission probabilities estimated based on these frequencies are given on the right side of the table (using the same $\vec{\alpha }$). For the sake of simplicity, we assume only the nucleotides G and T occur. Apart from this simplification and the restriction to four subtypes, this example is taken from actual HIV-1 sequences.

For the reasons just explained, we model the emission frequencies of the subtypes jointly (see Figure 1 for examples). In the following, we refer to subtypes being modeled jointly as sharing the same source. Moreover, an assignment of each subtype to its respective source is called a “source combination”. That is, if at a given site Subtypes A, C, G, and H are modeled by one source, B and K by another and D, F, and J by a third one, the assignment {*A*,*C*,*G*,*H*}→1, {*B*,*K*}→2, {*D*,*F*,*J*}→3 constitutes the source combination of the respective site. In a more general context, the source combinations are called the set of partitions (which play a role e.g. in determining the number of state-context trees of parsimonious higher-order HMMs [25]). Since the number of source combinations grows fast in the number of subtypes, we have to restrict the search space when determining the optimal source combination (see Subsection ‘Methods - Details on the information sharing protocol’).

In order to estimate the emission probabilities of the subtypes, we iterate through all possible source combinations for each position. For each source combination we estimate the emission probabilities of each source based on the nucleotide frequencies of the subtypes assigned to it using a Dirichlet prior^a^[7]. We obtain the emission probabilities of a subtype by averaging over all source combinations, taking for each source combination the emission probabilities of the source the subtype is assigned to. Hereby, we weight each source combination by its likelihood. Further details are provided in the Subsection ‘Methods - Details on the information sharing protocol’.

### Transition probabilities

In HIV, the distribution of gaps in the MSA provides little help in distinguishing different subtypes. Most parts of the genome are highly conserved, possessing nearly no insertions and deletions. In contrast, small parts are highly variable (possessing lots of gaps), but they vary so strongly that they provide little information for distinguishing different subtypes. For this reason, we estimate the transition probabilities (using a Dirichlet distribution as prior) for all subtypes jointly from the complete MSA. The same approach was applied by [7] for subfamilies belonging to the same protein family.

### jpHMM

**Figure 2:**
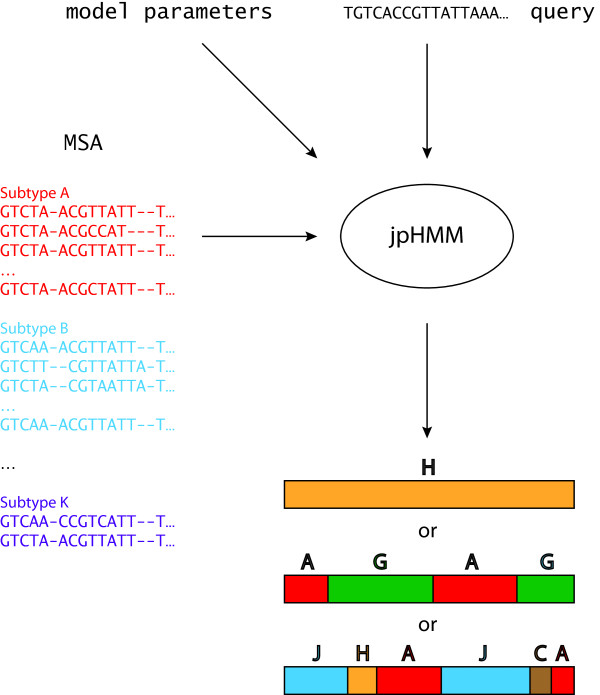
**Outline of the information flow of jpHMM.** As input, jpHMM expects an MSA partitioned into subtypes as well as an (unaligned) query sequence. Furthermore, the model parameters, based on which different details of the underlying model are determined, have to be set by the user. That is, the emission and transition probabilities and certain parts of the topology of the model are set based on the parameters. jpHMM then derives the most probable path through its pHMMs. As output, jpHMM assigns each position of the query sequence to a subtype of the MSA. Here, three possible outputs are shown.

**Figure 3:**
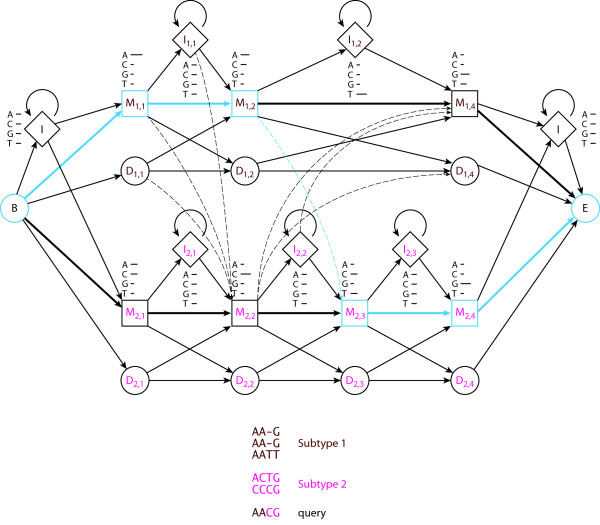
**The underlying model of jpHMM.** This model is illustrated using a toy example. It is built from a DNA MSA composed of two subtypes, with the first subtype having three sequences and the second one two sequences. For each match and insert state, a vector of emission probability values for the nucleotides is given. For the sake of clarity, the majority of transitions between the two subtypes is omitted. Moreover, the delete state directly right to the begin state B (from which one can go to each match state) as well as the delete state directly left to the end state E (to which one can go from each match state) were left out. High transition probabilities are represented by fat lines, low probabilities by thin lines, and the jumps between the subtypes by dashed lines. The Viterbi path with regards to the query sequence is colored in blue, i.e., the first two positions of the query are assigned to Subtype 1 and the last two to Subtype 2.

The recombination and breakpoint detection tool jpHMM requires a pre-calculated MSA of the HIV-1 subtypes as input (see Figure 2). Each subtype in the MSA is modeled by a separate pHMM (see Figure 3). In addition to the usual transitions within these pHMMs, the model allows for so-called jumps between different pHMMs at nearly any position in the MSA. That is, the model allows to jump between states associated with different subtypes, depending on the local similarity of the query sequence to the different subtypes. The complete model including the setting of the hyper-parameters is detailed in [4].

In order to determine the recombination pattern of a query sequence, the so-called Viterbi path [26] is computed, i.e., the most probable path of hidden states through the model generating this sequence. In this way, each position of the query sequence is assigned to exactly one parental subtype since i) each state of the model is assigned to exactly one pHMM (ignoring the special states at the begin and end of the model) and ii) each position of the query sequence is generated by one state of the model. The recombination breakpoints correspond to the positions of the jumps between different subtypes.

#### Details on the information sharing protocol

##### Notation

Let 1,…,*F* be the subtype (or, more generally, subfamily) indices. The individual sources in a source combination are indexed by 1,…,*R*. The space of all source combinations is denoted by S, the source of subtype *i* by *s*_*i*_. For each source *j* of a source combination $\vec{s}=\left\{s_{1},\ldots ,s_{F}\right\}$, we denote the subtypes assigned to source *j* by $\left\{i_{1}^{\left(j\right)},\ldots ,i_{m_{j}}^{\left(j\right)}\right\}$. That is, if *F*=4 and the subtypes 1, 2, and 4 are assigned to Source 1, and the Subtype 3 to Source 2, we have *m*_1_=3, *m*_2_=1, $\left\{i_{1}^{\left(1\right)},i_{2}^{\left(1\right)},i_{3}^{\left(1\right)}\right\}=\{1,2,4\}$, and $\left\{i_{1}^{\left(2\right)}\right\}=\{3\}$ (Notice that *R* and the $i_{k}^{\left(j\right)}$ are defined for a particular source combination, but that for the sake of readability we do not indicate that source combination by an additional index, in case several source combinations are considered). The number of nucleotides, denoted by *N*, is equal to 4.

##### Prior probability of number of sources

We denote the probability of a given number of sources *j* by *P*(*R* = *j*) = *ρ*_*j*_. These probabilities are estimated as described in [27]: For each column in the alignment of all full-length HIV-1 Group M sequences, we decide based on the AIC (Akaike Information Criterion) by how many sources the nucleotide frequencies of that column are modeled in the most likely way. Then, the prior probability of a given number of sources *R* is estimated by the relative frequency of for how many alignment columns *R* was found to be the most likely number of sources. Since the frequencies for *R*>6 are zero or near to zero, we set the maximum number of sources *R*_*max*_ to 6. The estimated values are (*ρ*_*j*_)_*j*=1,…, 6_=(0.37,0.30,0.20,0.097,0.029,0.0047).

##### Restricting the search space

**Figure 4:**
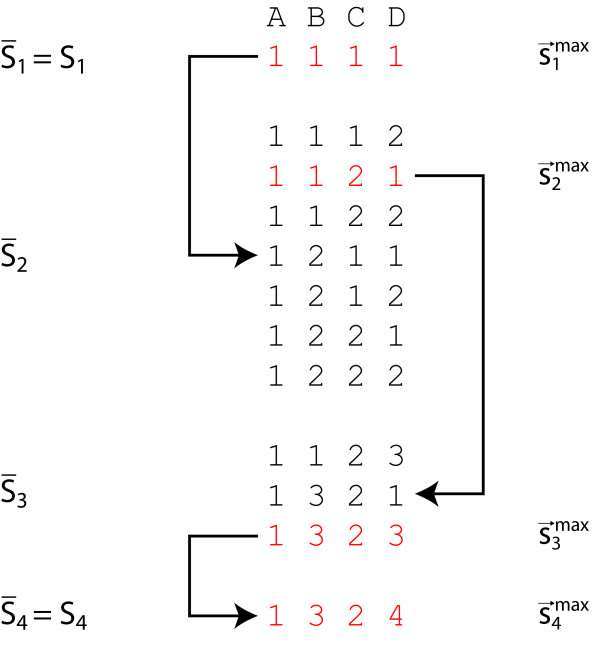
**Restricted search of the space of source combinations.** The heuristics described in Section ‘Details on the information sharing protocol - Restricting the search space’ is illustrated. In this example, we assume 4 subtypes A, B, C, and D. In each line a source combination is given, with the source combination in each ${\bar{S}}_{i}$ being grouped. The most likely source combinations in each $\bar{S_{i}}$ is colored in red. The arrows indicate how the spaces ${\bar{S}}_{1},\ldots ,{\bar{S}}_{4}$ are traversed during the search process.

In [27], we restricted our site-wise search in *S* to (*S*_*r*_)_*r*≤3_, where *S*_*r*_ is the space of source combinations composed of *r* sources. Since |*S*_*r*_|=*S*(*F*,*r*) (with *S*() the Stirling numbers of the second kind) and |*S*|=*B*(*F*) (with *B*() the Bell numbers^b^) a brute force search in the entire space *S* would imply a considerable computational burden: Increasing *R*_*max*_ from 3 to 6 would result in an increase of the computational effort by about a factor of 6. Hence, we restrict the search in *S* by the following procedure, illustrated in Figure 4. We search (*S*_*r*_)_*r*≤6_ successively, starting with *S*_1_, which only contains one source combination. Before searching *S*_*r*_, *r*≥2, we determine the most likely element of *S*_*r*−1_ (calling it ${\vec{s}}_{r-1}^{\text{max}}$). Then we restrict our search in *S*_*r*_ to those source combinations which can be obtained from ${\vec{s}}_{r-1}^{\text{max}}$ by dividing one of its sources into two, thus obtaining ${\vec{s}}_{r}^{\text{max}}$. We denote the subset of *S*_*r*_ in which we conduct the search by ${\bar{S}}_{r}$.

##### Estimation of emission probabilities

In case we only have one subtype, we use the following Bayesian approach to estimate the emission probabilities $\hat{\vec{p}}$ of the subtype at a fixed site given the nucleotide frequencies $\vec{n}$ at that site: We assume that the a priori distribution on the emission probabilities $\vec{p}$ is a Dirichlet distribution (see [7]), with parameter $\vec{\alpha }$ (estimated in [4]). The parameter may be interpreted as pseudo counts which are added to the nucleotide frequencies. The emission probabilities then are the corresponding relative frequencies of these modified nucleotide frequencies.

For the general case of more than one subtype, we here restrict ourselves to the estimation of the emission probability of nucleotide *ν*∈{1,…,*N*} of Subtype 1 for the sake of simplicity. We denote the probability to be estimated by ${\hat{p}}_{1,\nu }$, with the 1 being the index of the subtype. Without loss of generality, we can assume that this subtype is always assigned to Source 1. Moreover, we denote the nucleotide frequencies of subtype *i* at the position under consideration by ${\vec{n}}_{i}$ and the emission probabilities of source r by ${\vec{q}}_{r}$. Then, we estimate the emission probability of Subtype 1 by

(1) $$\begin{array}{ll} {\hat{p}}_{1,\nu } & =\overset{R_{\text{max}}}{\underset{r=1}{\sum }}\underset{\vec{s}\in S_{r}}{\sum }{\hat{p}}_{1,\nu }^{\left(s\right)}P(\vec{s}\;|{\vec{n}}_{1},\ldots {\vec{n}}_{F})\; \end{array}$$

with

() $$\begin{array}{lll} {\hat{p}}_{1,\nu }^{\left(s\right)} & =\int_{{\vec{q}}_{1}}q_{1,\nu }P\left({\vec{q}}_{1}\;|{\vec{n}}_{i_{1}^{\left(1\right)}},\ldots {\vec{n}}_{i_{m_{1}}^{\left(1\right)}},\vec{s}\right)d{\vec{q}}_{1}.\; & \; \end{array}$$

That is, we estimate the emission probability of Subtype 1 for each fixed source combination $\vec{s}$ by ${\hat{p}}_{1,\nu }^{\left(s\right)}$. Then we sum these estimates over all possible source combinations, weighting the summands by the probability of the respective source combination given the observed nucleotide frequencies.

We now consider the two factors in (1). In this, we assume that $\left\{i_{1}^{\left(1\right)},\ldots i_{m_{1}}^{\left(1\right)}\}=\{1,\ldots ,m\right\}$, i.e., Source 1 is composed of the subtypes 1 to *m*: 

1. In [27] (last equation on p. 6), we showed that

(2) $${\hat{p}}_{1,\nu }^{\left(s\right)}=\frac{\overset{m}{\underset{k=1}{\sum }}n_{k,\nu }+\alpha_{\nu }}{\overset{m}{\underset{k=1}{\sum }}|{\vec{n}}_{k}|+|\vec{\alpha }|}.$$

2. Applying Bayes’ rule, we obtain

(3) $$\begin{array}{lll} P\left(\vec{s}\;|{\vec{n}}_{1},\ldots ,{\vec{n}}_{F}\right) & =\frac{P\left({\vec{n}}_{1},\ldots ,{\vec{n}}_{F}|\vec{s}\right)P(\vec{s})}{P\left({\vec{n}}_{1},\ldots ,{\vec{n}}_{F}\right)}\; & \;\\ & =\frac{\overset{r}{\underset{l=1}{\prod }}P\left({\vec{n}}_{i_{1}^{\left(l\right)}},\ldots ,{\vec{n}}_{i_{m_{l}}^{\left(l\right)}}|\vec{s}\right)P(\vec{s})}{P\left({\vec{n}}_{1},\ldots ,{\vec{n}}_{F}\right)}\; \end{array}$$

By the first equation after Equation 4 in [27], we have

(4) $$\begin{array}{ll} P({\vec{n}}_{1},\ldots ,{\vec{n}}_{m}|\vec{s}) & =\left(\overset{m}{\underset{k=1}{\prod }}\frac{\Gamma (|{\vec{n}}_{k}|+1)}{\overset{N}{\underset{j=1}{\prod }}\Gamma (n_{k,j}+1)}\right)\\ & \;\times \frac{\Gamma (|\vec{\alpha }|)}{\overset{N}{\underset{j=1}{\prod }}\Gamma (\alpha_{j})}\frac{\overset{N}{\underset{j=1}{\prod }}\Gamma \left(\overset{m}{\underset{k=1}{\sum }}n_{k,j}+\alpha_{j}\right)}{\Gamma \left(\overset{m}{\underset{k=1}{\sum }}|{\vec{n}}_{k}|+|\vec{\alpha }|\right)}. \end{array}$$

Moreover, we introduce

(5) $$K=P({\vec{n}}_{1},\ldots ,{\vec{n}}_{F}),$$

as *K* is a constant independent of the source combination. In fact, we do not need to calculate *K* because we can make use of the fact that

$$\overset{R_{\text{max}}}{\underset{r=1}{\sum }}\underset{\vec{s}\in S_{r}}{\sum }P(\vec{s}\;|{\vec{n}}_{1},\ldots ,{\vec{n}}_{F})=1.$$

Finally, we assume that all source combinations composed of the same number of sources share the same prior probability, i.e.,

(6) $$P(\vec{s})=\frac{\rho_{r}}{\left|S_{r}\right|},$$

Now, plugging (2), (4), (5), and (6) into (1), we obtain

(7) $$\begin{array}{ll} {\hat{p}}_{1,\nu } & =\frac{1}{K}\overset{R_{\text{max}}}{\underset{r=1}{\sum }}\frac{\rho_{r}}{\left|S_{r}\right|}{\left(\frac{\Gamma (|\vec{\alpha }|)}{\overset{N}{\underset{j=1}{\prod }}\Gamma (\alpha_{j})}\right)}^{r}\underset{\vec{s}\in S_{r}}{\sum }\left(\overset{r}{\underset{l=1}{\prod }}\phi_{\nu ,l}^{\left(r\right)}\right)\\ & \;\times \frac{\overset{m_{1}}{\underset{k=1}{\sum }}n_{i_{k}^{\left(1\right)},\nu }+\alpha_{\nu }+1}{\overset{m_{1}}{\underset{k=1}{\sum }}|{\vec{n}}_{i_{k}^{\left(1\right)}}|+|\vec{\alpha }|+1} \end{array}$$

with

(8) $$\begin{array}{c} \phi_{\nu ,l}^{\left(r\right)}=\left(\overset{m_{l}}{\underset{k=1}{\prod }}\frac{\Gamma \left(|{\vec{n}}_{i_{k}^{\left(l\right)}}|+1\right)}{\overset{N}{\underset{j=1}{\prod }}\Gamma \left(n_{i_{k}^{\left(l\right)},j}+1\right)}\right)\frac{\overset{N}{\underset{j=1}{\prod }}\Gamma \left(\overset{m_{l}}{\underset{k=1}{\sum }}n_{i_{k}^{\left(l\right)},\;j}+\alpha_{j}\right)}{\Gamma \left(\overset{m_{l}}{\underset{k=1}{\sum }}|{\vec{n}}_{i_{k}^{\left(l\right)}}|+|\vec{\alpha }|\right)}. \end{array}$$

**Figure 5:**
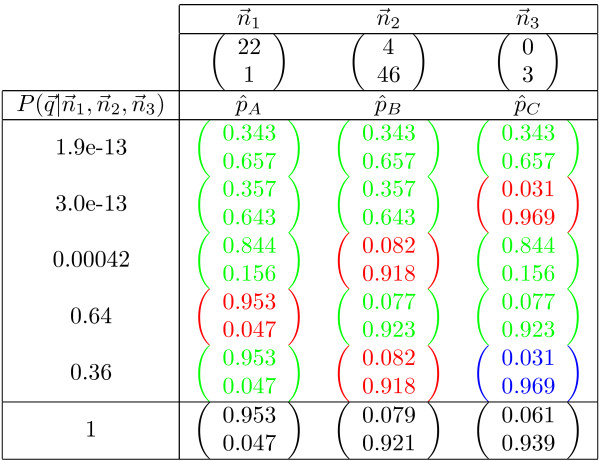
**Detailed calculation of the emission probabilities.** Simplified example of the detailed derivation of the emission probabilities from the nucleotide frequencies. At the top, the nucleotide frequencies for each subtype are given and, at the bottom, the emission probabilities derived from these frequencies are provided. In between, for each source combination, the probability of the respective source combination given the nucleotide frequencies is shown on the left side, whereas the emission probabilities of each subtype for the particular source combination is given on the right side. The coloring indicates to which source each subtype belongs for the respective source combination. For example, the third column in the middle block represents the source combination, for which Subtype A and C belong to one source (green) and Subtype B to another (red), yielding emission probabilities of 0.844 and 0.156, respectively, for the green source and of 0.082 and 0.918, respectively, for the red one (we elaborate on some details in this table in the supplements). The simplification made consists of the restriction to 3 subtypes and 2 nucleotides.

With these two formulas, ${\hat{p}}_{\nu }$ is calculable using only known quantities. An example illustrating the details on the calculation of the emission probabilities is given in Figure 5. We provide further details on the calculations presented in this subsection in the Additional file 1: Supplements.

## Results

### Test data

**Figure 6:**
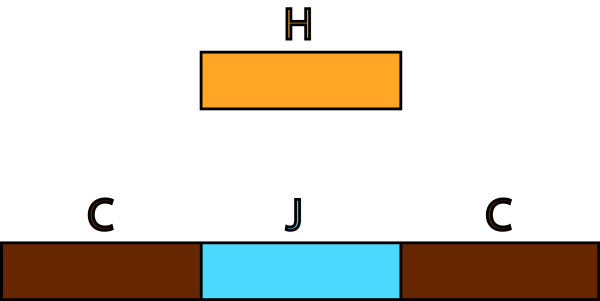
**Example sequences of the test data.** Sequences of the test dat sets *T*_*pure*_ (upper) and *T*_*rec*_ (lower) are shown.

We evaluate our algorithm on two sets of test sequences, *T*_*rec*_ and *T*_*pure*_ (see Figure 6).

The sequences in *T*_*rec*_ are semi-artificial recombinants of real-world subtype sequences. The purpose of *T*_*rec*_ is to test the sensitivity of our algorithm with respect to the detection of segments originating from small-size subtypes. The sequences in *T*_*rec*_ are composed of three segments, each of length 250 bps. The first and last segment stem from a regular-size subtype (A-D, F, and G), the middle part stems from one of the small-size subtypes (H, J, and K). The choice to group the subtypes this way into normal- and small-size ones, is canonical since the subtypes H, J, and K contain only 2 resp. 3 sequences, whereas the other subtypes contain 30+ sequences. For each pair composed of a normal- and a small-size subtype, *T*_*rec*_ contains 10 semi-artificial recombinants composed of sequences from the respective pair of subtypes. That is, we have 180 sequences in total. The first position of the 750 bps long genome part from which a semi-artificial recombinant is constructed is chosen randomly between position 1007 and 8501 with respect to the HIV-1 reference sequence HXB2 (see [28]).

The set *T*_*pure*_ is composed of parts of sequences from regular-size pure subtypes, i.e. partial subtype sequences. The purpose of *T*_*pure*_ is to test the specificity of our algorithm with respect to the detection of segments originating from small-size and regular-size subtypes. Each sequence in *T*_*pure*_ has a length of 2000 bps. For each subtype, *T*_*pure*_ contains 20 partial sequences, i.e. 180 sequences in total. The starting position of the sequence part taken is chosen randomly between position 1007 and 8501 with respect to HXB2.

### Performance measures

**Figure 7:**
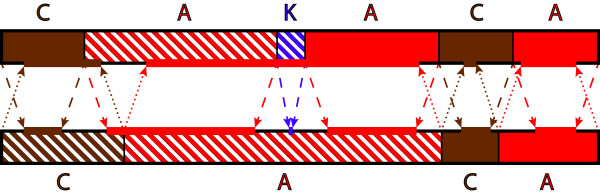
**Calculation of*s***_***segm***_** between two subtype-wise segmentations.** The arrows show how the segments of both classifications are trimmed. Hereby, the colored bar between two arrows on the bottom side of the upper classification indicates which positions of the upper classification have to be assigned to a subtype as given by the bar such that the corresponding segment of the lower classification is counted as conforming to the upper classification (same applies with the roles of the lower and upper classification being switched). Segments which do not conform to the other classification are shaded.

We evaluate the performance of our algorithm by two measures: i) the fraction *s*_*pos*_ of sequence positions correctly classified, ii) the conformance *s*_*segm*_ of the predicted and the correct subtype pattern. Hereby, *s*_*segm*_ is computed like follows (see Figure 7). All segments of the correct subtype pattern are trimmed by 50 bps on both ends. If a segment is too short for this procedure, only its middle point is kept. Then each of these trimmed segments is checked whether all its position coincide with the subtype in the predicted pattern. The same is done with the roles of the correct and the predicted pattern switched. The score *s*_*segm*_ is then defined as the fraction of all trimmed segments which match their counterpart.

The motivation for using the two measures *s*_*pos*_ and *s*_*segm*_ consists in each concentrating on a different type of classification error: the primary purpose of *s*_*pos*_ is to identify smaller shifts of the breakpoints, under the assumption that the predicted recombination patterns are correct. On the contrary, *s*_*segm*_ does not aim for these errors, but is designed for detecting different recombination patterns. Furthermore, these scores are suitable for being used in an automatic evaluation. In contrast to that, using the distances between the correct and the predicted breakpoints (which is a more canonical approach) would require to deal with differences in the recombination patterns (like the recombination patterns AGA and AGAGA) manually. We use the arithmetic mean of *s*_*pos*_ and *s*_*segm*_ as an overall score for a single test sequence, denoting it *s*_*tot*_.

Notice that applying the introduced measures on the two test sets does not exactly measure what we stated as purpose of the test sets (sensitivity resp. specificity to detect segments originating from small-size subtypes): For *T*_*rec*_ we also consider misclassification in the first and last segment and for *T*_*pure*_ we do not distinguish between wrongful inserts of segments of normal- and small-size subtypes. One can rather view *T*_*rec*_ and *T*_*pure*_ as boundaries between which all reasonable application scenarios lie: No standard user will want to emphasize sensitivity more than measured by one of the introduced scores on *T*_*rec*_ and, analogously, no one specificity more than measured on *T*_*pure*_.

### Versions of jpHMM

We compare four different versions of jpHMM, as summarized in Table 2. Since we have already shown in [29] that the performance of jpHMM can be improved by jointly modeling the transition probabilities for all subtypes, we do not consider the original version of jpHMM in our evaluation, which models them separately for each subtype. We compare four approaches to estimate the emission probabilities: i) jpHMM ^*prob*^: the probabilistic model utilizing an information sharing protocol introduced in this paper, ii) jpHMM ^*ml*^: the regularized linear discriminant learning presented in [29], iii) jpHMM ^*scal*^: a heuristic approach scaling the pseudocounts depending on the number of sequences in a subtype already evaluated in [29], and iv) jpHMM ^*semi*^: the original algorithm introduced in [4].

**Table 2:** The evaluated models

**Abbreviation**	**Emission**	**Transition**	**Evaluated**
	**probabilities**	**probabilities**	
jpHMM ^*prob*^	Probabilistic model	Joint	*✓*
jpHMM ^*ml*^	Machine learning	Joint	*✓*
jpHMM ^*scal*^	Pseudocount scaling	Joint	*✓*
jpHMM ^*semi*^	Original	Joint	*✓*
jpHMM ^*orig*^	Original	Separate	

The first column gives the abbreviation used for the respective model in what follows. The second column indicates how the emission probabilities are modeled: the probabilistic model introduced in this paper, the machine learning approach presented in [29], a scaling the pseudocounts depending on subtype size evaluated in [29], or the original algorithm introduced in [4]. The third column shows whether the transition probabilities are modeled jointly or separately. The last column indicates whether the respective approach was evaluated in this paper.

#### Comparison of different versions of jpHMM

**Figure 8:**
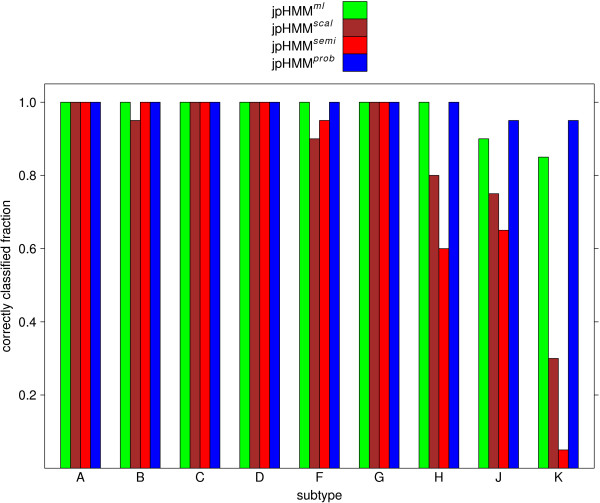
**The fraction of correctly classified sequences for*T***_***pure***_**.** This fraction is shown for the application of jpHMM ^*ml*^, jpHMM ^*scal*^, jpHMM ^*semi*^, and jpHMM ^*prob*^ to *T*_*pure*_, stratified by subtypes.

**Figure 9:**
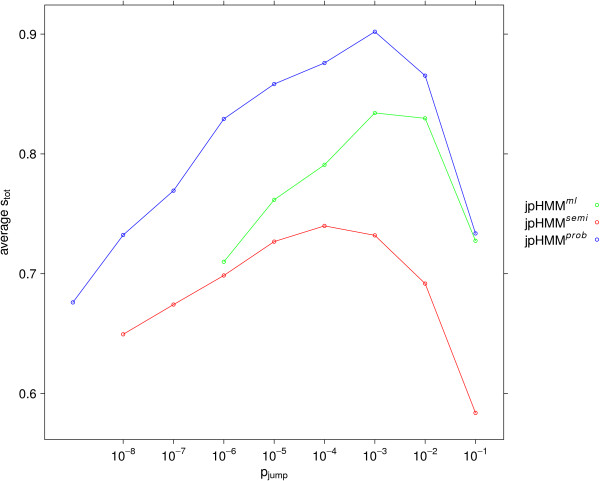
**The performance of different versions of jpHMM on*T***_***rec***_**.** The performance of jpHMM ^*ml*^, jpHMM ^*semi*^, and jpHMM ^*prob*^ on *T*_*rec*_ for different choices of *p*_*jump*_ is shown. It is measured by *s*_*tot*_.

**Figure 10:**
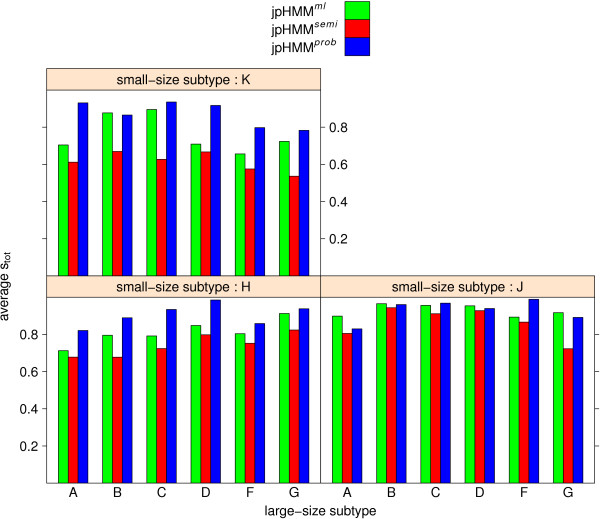
**The stratified performance of different versions of jpHMM on*T***_***rec***_**.** The performance (measured in *s*_*tot*_) of jpHMM ^*ml*^, jpHMM ^*semi*^, and jpHMM ^*prob*^ on *T*_*rec*_ is shown, with the semi-artificial recombinant sequences *T*_*rec*_ being stratified by which small- and which large-size subtype was used for the generation of the respective sequence.

We compare the four jpHMM versions jpHMM ^*semi*^, jpHMM ^*scal*^, jpHMM ^*ml*^, and jpHMM ^*prob*^ on the test sequence set *T*_*pure*_ and all of them but jpHMM ^*scal*^ on *T*_*rec*_. The results of this evaluation are illustrated in Figures 8, 9 and 10. jpHMM ^*scal*^ is not tested on *T*_*rec*_ since the purpose of introducing jpHMM ^*scal*^ was to demonstrate that it is not possible to reach the performance of jpHMM ^*ml*^ or jpHMM ^*prob*^ by employing an arbitrary heuristic approach to estimate the emission probabilities. For this purpose, testing only on *T*_*pure*_ is sufficient.

On *T*_*pure*_, jpHMM ^*semi*^ classifies 82.2% of the sequences correctly, jpHMM ^*scal*^ 85.6%, jpHMM ^*ml*^ 97.2%, and jpHMM ^*prob*^ 98.9%. Applying the sign test yields that jpHMM ^*prob*^ is better than jpHMM ^*semi*^ with p-value *p*=9·10^−10^, than jpHMM ^*scal*^ with *p*=6·10^−8^, and than jpHMM ^*ml*^ with *p*=0.125.

On *T*_*rec*_, jpHMM ^*semi*^ achieves an average *s*_*tot*_ of 0.74, jpHMM ^*ml*^ of 0.83, and jpHMM ^*prob*^ of 0.90. Based on the Wilcoxon signed rank test jpHMM ^*prob*^ performs better than jpHMM ^*semi*^ with *p*<2·10^−16^ and outperforms jpHMM ^*ml*^ with *p*=3·10^−6^.

#### Comparison of jpHMM with Simplot and Bootscanning

##### Setting

Simplot employs a distance-based method, whereas Bootscanning is based on a phylogenetic approach. Both can be applied to both single sequences and groups of sequences, such as HIV-1 subtypes. Simplot as well as Bootscanning require a certain number of sequences (respectively, groups) as input (three for Simplot, four for Bootscanning, including the query sequence).

As neither Simplot nor Bootscanning allow for automatic execution, we restrict our comparison to 84 sequences of *T*_*pure*_ and 36 sequences of *T*_*rec*_. Since it turned out during our evaluation that correctly classifying segments from large subtypes is not a challenge for any algorithm, we compared the methods i) for each large-size subtype on four randomly selected sequences from *T*_*pure*_ and ii) on all sequences of *T*_*pure*_ from small-size subtypes. For the comparison on semi-artificial recombinants, we randomly picked two sequences for each combination of a large- and a small-size subtype from *T*_*rec*_.

##### Results

All four methods classify the 24 pure segments from large-size subtypes correctly. Out of the 60 segments from the small-size subtypes, jpHMM ^*prob*^ misclassifies 2, jpHMM ^*ml*^ 5, Simplot 11, and Bootscanning 6. Applying the sign test yields that jpHMM ^*prob*^ performs better than Simplot with *p*=0.006 and better than Bootscanning with *p*=0.11.

On the 36 semi-artificial recombinants taken from *T*_*rec*_, jpHMM ^*prob*^ achieves an average *s*_*pos*_ of 0.926, jpHMM ^*ml*^ of 0.863, Simplot of 0.833, and Bootscanning of 0.839. Hereby, jpHMM ^*prob*^ achieves a higher score than Simplot for 28 sequences with Simplot better performing for the remaining 8 sequences. Comparing jpHMM ^*prob*^ with Bootscannnig, jpHMM ^*prob*^ performs better in 32 out of 36 cases, with Bootscanning being better for the rest. Applying the Wilcoxon signed-rank test yields that jpHMM ^*prob*^ performs better than Simplot with *p*=2·10^−4^ and better than Bootscanning with *p*=2·10^−6^.

#### Influence of the degree of conservation on the performance of jpHMM

**Figure 11:**
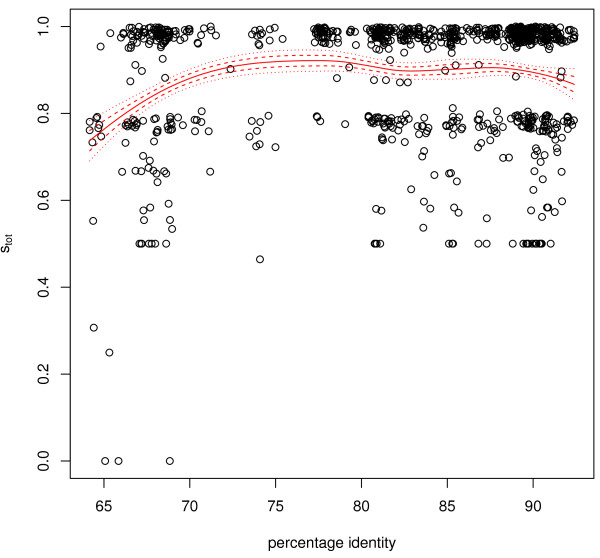
**The influence of the degree of conservation on the performance of jpHMM **^***prob***^**.** The performance of jpHMM ^*prob*^ (measured in *s*_*tot*_) on semi-artificial recombinants against the pairwise percentage identity of the sequences in the genome region from which the respective semi-artificial recombinant was taken. The LOESS curve together with its standard deviation (dashed) and the double of the standard deviation (dotted) is given as red line.

In order to measure the influence of the variability of the genome part from which a sequence stems on the performance of jpHMM ^*prob*^, we evaluate the performance of jpHMM ^*prob*^ on semi-artificial recombinants. Since the results from pure sequences are less conclusive, we restrict our evaluation to recombinants. As *T*_*rec*_ is too small to achieve significant results in this regards, we employ an extended version of *T*_*rec*_. Instead of 10 sequences per subtype pair we use 50. We measure the degree of conservation by the pairwise percentage identity of all sequences from our input MSA in the genome region from which the respective semi-artificial recombinant was taken. The results are shown in Figure 11.

The degree of conservation has an influence on the performance of jpHMM ^*prob*^. The score achieved by jpHMM ^*prob*^ for sequences with identity lower than 70% is significantly lower than the one for sequences with identity higher than 70%. Above the threshold of 70%, the degree of conservation does not seem to have a significant influence on the prediction performance.

#### Application to CRF04

**Figure 12:**
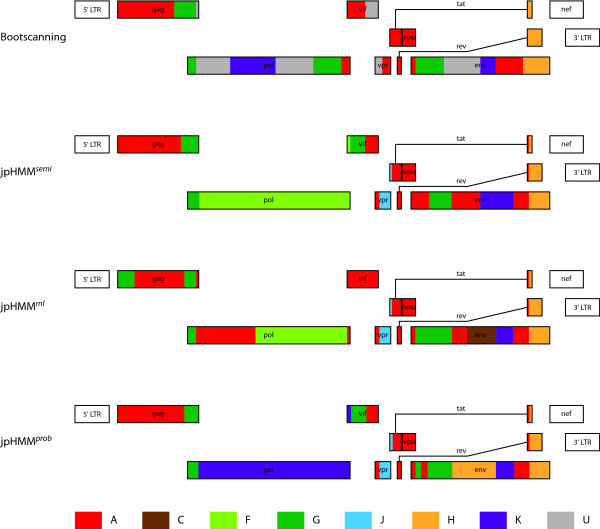
**The classification of CRF04.** The classifications as given on the LANL website (first) and provided by jpHMM ^*semi*^ (second), jpHMM ^*ml*^ (third), and jpHMM ^*prob*^ (last), respectively. Here, Subtype U stands for an unknown subtype.

In order to verify that jpHMM ^*prob*^ is capable of handling full-length sequences which exhibit a recombination pattern observed in a real recombinant, we apply jpHMM ^*semi*^, jpHMM ^*prob*^, and jpHMM ^*ml*^ to the sequence AF119819 classified as recombinant of type CRF04 [30,31] in the LANL database. CRF04 is one of the CRFs whose genome allegedly stems in part from a small-size subtype (subtypes H and K for CRF04). The segmentation of CRF04 provided in the LANL database was obtained by Bootscanning. Since for this kind of application, one normally does not want the subtype-wise segmentation to get too fragmented, we use *p*_*jump*_=10^−8^ for jpHMM ^*semi*^, *p*_*jump*_=10^−6^ for jpHMM ^*prob*^ and *p*_*jump*_=10^−7^ for jpHMM ^*ml*^. We here have to use a considerably smaller jump probability for jpHMM ^*semi*^ than for jpHMM ^*prob*^ since we have to smooth out the misclassifications jpHMM ^*semi*^ does due to its less performant estimator of the emission probabilities. The results and the classification given on the LANL website are given in Figure 12.

Notice that subtypes F and K as well as subtypes A, G, and J, respectively, are closely related [32]. Hence, one can expect different algorithms to classify a particular segment as belonging to two different subtypes of one of these two phylogenetic groups. Furthermore, the original classification assigns parts of the sequence to Subtype U (“unknown”). Since the input alignment of jpHMM does not contain sequences of an unknown subtype, jpHMM cannot designate any part of a query sequence as Subtype U.

We also explored how the results of jpHMM ^*prob*^ change with different parameter values. Increasing *p*_*jump*_ to 10^−5^ does not change the prediction at all, while for *p*_*jump*_=10^−4^ the number of segments is increased by four. Decreasing *p*_*jump*_ to 10^−7^ or 10^−8^ yields the same result. The fragment from Subtype G, predicted in the vif gene, vanishes and instead Subtype A is predicted in this location.

#### Running time

On an Intel Core 2 Duo E8400 with 3 GHz and 4 GB of RAM, the application of jpHMM ^*prob*^ to a sequence from *T*_*rec*_ (length 750 bps) takes about 16 minutes. The lion’s share of the computational time is due to the calculation of the emission probabilities. It could be decreased considerably by implementing further heuristics to neglect source combinations with very low likelihood.

## Discussion and conclusions

In this paper, we presented a novel information sharing protocol, realized in the form of a probabilistic model, for estimating the emission probabilities of pHMMs. We incorporated this protocol into the recombination detection program jpHMM and applied it to semi-artificial and real-world HIV-1 Group M sequences. As we were able to confirm an improvement of the prediction accuracy of jpHMM by our protocol, we demonstrated the value of the protocol for a crucial biological and medical application employing pHMMs.

### Biological/medical relevance

The work of biological and medical researchers can be supported in various ways by improving the precision of recombination and breakpoint detection for HIV-1. For example, discovering recombination-free genome regions allows for the identification of barriers to genetic crossing between different viral strains. This kind of barriers then may help identifying vulnerable aspects of HIV biology in form of combinations of mutations strongly decreasing the survivability of an HI virus. This can facilitate finding promising targets for antiviral strategies (Talk of M. Negroni on HIV Dynamics & Evolution 2011).

Furthermore, recombination analysis and breakpoint detection do not only play a role for numerous viruses, e.g., flaviviruses, coronaviruses, alphaviruses, avian oncoviruses, rotaviruses, and influenza viruses [23,33-39]. It is also needed for bacteria [40,41] and within the scope of the detection of 16S rRNA chimera, which evolve during PCR [42-44]. Moreover, our approach could also be employed to improve software for remote homology detection of proteins. A heuristic probabilistic approach addressing the problem of small protein families has been introduced in [3].

### Testing

We have applied i) the novel version of jpHMM incorporating our information sharing protocol, ii) former versions of jpHMM, and iii) the established recombination breakpoint detection tools Simplot and Bootscanning to i) sequences designated (in the LANL HIV database) as originating from a pure subtype, ii) semi-artificial recombinants of two real-world subtype sequences, iii) a full-length sequence of CRF04. In total, we compared the novel version of jpHMM with three formerly introduced ones, differing with regards to how they estimate the emission probabilities: i) a machine learning approach using linear discriminant learning, ii) a heuristic approach of scaling the pseudocounts, iii) the original approach without adaptions for small-size subtypes.

The results were compared using appropriate performance measures. On all test sequence sets, the novel version of jpHMM either significantly outperformed the other algorithms or achieved better results, but without reaching statistical significance. The latter is mostly a result of the fact that for most algorithms the task of correctly classifying segments of large-size subtypes is not a challenge, thus yielding very similar results for the corresponding type of test.

Compared with the machine-learning approach (jpHMM ^*ml*^) introduced in [29], the method presented in this paper (jpHMM ^*prob*^) seems to be considerably better performing on very small subtypes: On the one hand, jpHMM ^*ml*^ achieves an average score (*s*_*tot*_) of 0.87 on the semi-artificial recombinants constructed from Subtype H or J, respectively, whereas the average score on recombinants constructed from Subtype K is 0.76. That is, there is a difference in regard to the average score of 0.11 between recombinants built from Subtype H and J, respectively, and Subtype K. On the other hand, for jpHMM ^*prob*^ the average score is 0.92 for recombinants built from Subtype H and J, respectively, and 0.87 for Subtype K. That is, a difference of 0.05 can be observed. Thus, our model-driven information sharing approach seems in particular more suitable to model very small subtypes than a machine learning approach based on regularized linear discriminant learning and a transformation of the resulting weights into valid probabilities via a softmax function.

Astonishingly, for the tests on semi-artificial recombinants, the phylogenetic distance between the respective small- and large-size subtypes does not seem to have a notable influence on the performance of jpHMM ^*prob*^. The phylogenetically nearest large-size subtype of Subtype H is Subtype C, of Subtype J is Subtype A, and of Subtype K is Subtype F. Nevertheless, only semi-artificial recombinants composed of Subtype J and A achieve considerably poorer results than recombinants composed of Subtype J and another large-size subtype. For Subtypes H and K, recombinants built from Subtypes C and F, respectively, do not yield a notably low performance. Since Subtypes K and F are phylogenetically closer than H and C (J and A, respectively), we can conclude that the low performance for recombinants built from Subtype J and A is probably simply coincidental and that the influence of the phylogenetic distance is at most weak for recombinants between HIV-1 Group M subtypes.

The relatively weak performance of jpHMM ^*prob*^ on partial sequences stemming from weakly conserved genome parts can easily be explained by the fact that jpHMM is not well suited for this kind of sequences: pHMMs assume that the group of sequences to be modeled can be well aligned. If not, the resulting pHMM consists mostly of insert states which do not constitute a sophisticated model for unalignable sequences.

One might wonder whether our test setting, with very short segments, is appropriate if the normal application requires considerably lower jumping probabilities. That is, one could suppose that our test setting does not prove that the algorithm performs well in correctly classifying a very long fragment (i.e. several thousand bps) stemming entirely from one subtype. But if the algorithm tended to assign such a fragment to more than one subtype, it would also misclassify shorter subfragments. Hence, by testing it on partial length sequences from one subtype we have in fact also covered these long fragments from one subtype.

### Application to CRF04

In order to verify that jpHMM ^*prob*^ is capable of handling real world data, we applied it to a CRF04 sequence. This application shows – to some extent – why we stuck to semi-artificial recombinants instead of evaluating jpHMM ^*prob*^ on CRFs. For CRFs, it is unclear which classification is the “correct” one. This results from the scientific community not coming to any consensus on which subtype classifications are the correct ones. Beyond that, it is even discussed whether the entire classification system exhibits flaws: For example, it has been debated whether Subtype G is in fact a recombinant and CRF02 a pure subtype [45-48]. Moreover, the total number of CRFs is still quite small and a large fraction of them possesses only a few breakpoints, i.e., the number of potential test sequences is limited. Furthermore, it is a widespread approach to evaluate the performance of recombination detection tools using (semi-)artificial recombinants [21,46,49,50]. Therefore, we chose to test jpHMM ^*prob*^ on semi-artificial recombinants, despite one could perceive testing on CRFs as more “realistic”.

### Outlook

We recently have developed a probabilistic algorithm called the “Unknown Subtype Finder (USF)” [27] for the purpose of automatically detecting parts of an input sequence which stem from a subtype yet unknown. For USF, the emission probabilities of an unknown subtype are also calculated employing the concept of sources which is the base of the information sharing protocol presented in this paper. Consequently, we plan the integration of USF and jpHMM into one tool. This tool then could be used to assign the known subtypes of HIV-1 to a (full- or part-length) query sequence as well as to detect segments of the genome originating from a subtype yet unknown.

Beyond this, one might think of several extensions to the probabilistic model presented in this paper: 

• The probability of whether the emission probabilities of a given set of subtypes are modeled jointly might depend on the subtypes in the set. Like this, it could be accounted for the fact that phylogenetic close subtypes should tend more to be modeled jointly than phylogenetic distant ones. As the phylogeny of the subtypes, which subtypes tend to be jointly modeled might change along the genome.

• The information sharing protocol could be based on a hierarchical classification system (as it is with protein families, superfamilies, folds, and classes [51]) instead of a flat classification system (for example, HÌV-1 Group M subtypes). Similarly, how strongly the emission probabilities of an entity from the classification system (e.g. a protein family) is influenced by another entity could depend on how close the two entities are in the classification system. Analogously, one could also allow for unknown entities differing with regards to how distant they are to the known entities, e.g., unknown protein families and unknown protein superfamilies.

• The probability of jumping between subtypes could be modeled as varying along the genome, motivated by the fact that the distribution of recombination breakpoints is not uniform among the genome [52].

## Availability

The C++ source code of jpHMM and Matlab code for evaluating the different versions of jpHMM is available at http://jphmm.gobics.de.

## Endnotes

^a^ The explanation here emphasizes understandability. Due to computational efficiency, the actual computations are carried out in a quite different way.

^b^ The first ten Bell numbers *B*(1) through *B*(10) are 1, 2, 5, 15, 52, 203, 877, 4140, 21147, 115975.

## Competing interests

The authors declare that they have no competing interests.

## Authors’ contributions

IB conceived the approach and developed, implemented, and tested the algorithm. AKS carried out modifications on jpHMM. CC provided statistical expertise. TM and FS assisted in the data handling. All authors read and approved the final manuscript.

## Supplementary Material

Additional file 1 Supplements. Click here for file
