# Supplementary material for: A model-based information sharing protocol for profile Hidden Markov Models used for HIV-1 recombination detection
Source: BMC Bioinformatics. 2014 Jun 19;15:205. doi: 10.1186/1471-2105-15-205 (PMC4230192; doi:10.1186/1471-2105-15-205)
Supplement: Additional file 1 — Supplements. [file 1471-2105-15-205-S1.pdf]

1 Estimation of emission probabilities

In this section we show in detail how to derive (7) and (8) in the article: Plugging first (5) and (6) and then (4) into (3) yields

$$
\begin{aligned}
& P(\vec{s}|\vec{n}_1,\ldots,\vec{n}_F) \\
= \; & \frac{\rho_r}{|S_r|K}\prod_{l=1}^{r} P(\vec{n}_{i_1^{(l)}},\ldots,\vec{n}_{i_{m_l}^{(l)}}|\vec{s}) \\
= \; & \frac{\rho_r}{|S_r|K}\prod_{l=1}^{r}\left[\left(\prod_{k=1}^{m_l}\frac{\Gamma(|\vec{n}_{i_k^{(l)}}|+1)}{\prod_{j=1}^{N}\Gamma(n_{i_k^{(l)},j}+1)}\right)\frac{\Gamma(|\vec{\alpha}|)}{\prod_{j=1}^{N}\Gamma(\alpha_j)}\frac{\prod_{j=1}^{N}\Gamma(\sum_{k=1}^{m_l}n_{i_k^{(l)},j}+\alpha_j)}{\Gamma(\sum_{k=1}^{m_l}|\vec{n}_{i_k^{(l)}}|+|\vec{\alpha}|)}\right] \\
= \; & \frac{\rho_r}{|S_r|K}\left(\frac{\Gamma(|\vec{\alpha}|)}{\prod_{j=1}^{N}\Gamma(\alpha_j)}\right)^r\prod_{l=1}^{r}\left[\left(\prod_{k=1}^{m_l}\frac{\Gamma(|\vec{n}_{i_k^{(l)}}|+1)}{\prod_{j=1}^{N}\Gamma(n_{i_k^{(l)},j}+1)}\right)\frac{\prod_{j=1}^{N}\Gamma(\sum_{k=1}^{m_l}n_{i_k^{(l)},j}+\alpha_j)}{\Gamma(\sum_{k=1}^{m_l}|\vec{n}_{i_k^{(l)}}|+|\vec{\alpha}|)}\right].
\end{aligned}
$$

Then, applying this and (2) with $\vec{s}$ being of the general form, i.e.

$$
\hat{p}_{1,\nu}^{(s)} = \frac{\sum_{k=1}^{m_1} n_{i_k^{(1)},\nu}+\alpha_\nu+1}{\sum_{k=1}^{m_1}|\vec{n}_{i_k^{(1)}}|+|\vec{\alpha}|+1},
$$

to (1), we obtain

$$
\begin{aligned}
& \hat{p}_{1,\nu} \\
= \; & \sum_{r=1}^{R_{max}}\sum_{\vec{s}\in S_r}\frac{\sum_{k=1}^{m_1} n_{i_k^{(1)},\nu}+\alpha_\nu+1}{\sum_{k=1}^{m_1}|\vec{n}_{i_k^{(1)}}|+|\vec{\alpha}|+1}\frac{\rho_r}{|S_r|K}\left(\frac{\Gamma(|\vec{\alpha}|)}{\prod_{j=1}^{N}\Gamma(\alpha_j)}\right)^r \times \\
& \qquad \prod_{l=1}^{r}\left[\left(\prod_{k=1}^{m_l}\frac{\Gamma(|\vec{n}_{i_k^{(l)}}|+1)}{\prod_{j=1}^{N}\Gamma(n_{i_k^{(l)},j}+1)}\right)\frac{\prod_{j=1}^{N}\Gamma(\sum_{k=1}^{m_l}n_{i_k^{(l)},j}+\alpha_j)}{\Gamma(\sum_{k=1}^{m_l}|\vec{n}_{i_k^{(l)}}|+|\vec{\alpha}|)}\right] \\
= \; & \frac{1}{K}\sum_{r=1}^{R_{max}}\frac{\rho_r}{|S_r|}\left(\frac{\Gamma(|\vec{\alpha}|)}{\prod_{j=1}^{N}\Gamma(\alpha_j)}\right)^r \times \\
& \qquad \sum_{\vec{s}\in S_r}\left\{\prod_{l=1}^{r}\left[\left(\prod_{k=1}^{m_l}\frac{\Gamma(|\vec{n}_{i_k^{(l)}}|+1)}{\prod_{j=1}^{N}\Gamma(n_{i_k^{(l)},j}+1)}\right)\frac{\prod_{j=1}^{N}\Gamma(\sum_{k=1}^{m_l}n_{i_k^{(l)},j}+\alpha_j)}{\Gamma(\sum_{k=1}^{m_l}|\vec{n}_{i_k^{(l)}}|+|\vec{\alpha}|)}\right]\right\}\frac{\sum_{k=1}^{m_1} n_{i_k^{(1)},\nu}+\alpha_\nu+1}{\sum_{k=1}^{m_1}|\vec{n}_{i_k^{(1)}}|+|\vec{\alpha}|+1},
\end{aligned}
$$

with the term in brackets being $\phi_{\nu,l}^{(r)}$.

2 Last two source combinations in Table 4

One might wonder why the fourth source combination achieves a higher probability than the fifth one for the following reason: The fifth source combination assigns Subtype B and C to different sources, whereas the fourth one does not. Hence, the emission probabilities of the fifth source combination fit the nucleotide frequencies better. That is, emission probabilities of (0.082, 0.918) and (0.031, 0.969), respectively, correspond

better to nucleotide frequencies of (4, 46) and (0, 3), respectively, than using joint emission probabilities of (0.077, 0.923) for both. Hence, one could expect a higher probability for the fifth source combination.

This phenomenon can be explained by the following toy example: Let us assume the nucleotide frequencies were both (0, 1) instead of (4, 46) and (0, 3) and let us assume that $\alpha = (1, 1)$, i.e., a flat prior for $\vec{p}$. Applying the same reasoning, one would then expect the probabilities for the fourth and fifth source combination to be about the same since one does not loose or gain much by modeling Subtype B and C together or separately. But in fact, neglecting Subtype A, the probabilities of the fourth and fifth, respectively, source combination are $\frac{1}{3}$ and $\frac{1}{4}$, respectively. To understand this, we have to look at the probabilities

$$P(\vec{n}_B, \vec{n}_C | \vec{s} = (1, 1)) = \int_{\vec{p}} P(\vec{n}_B | \vec{p}) P(\vec{n}_C | \vec{p}) d\vec{p} = \frac{1}{3} \quad (1)$$

and

$$P(\vec{n}_B, \vec{n}_C | \vec{s} = (1, 2)) = \int_{\vec{p}} P(\vec{n}_B | \vec{p}) d\vec{p} \cdot \int_{\vec{p}} P(\vec{n}_C | \vec{p}) d\vec{p} = \frac{1}{4}, \quad (2)$$

which is basically the first equation after (4) in [1]. Since $P(\vec{n}_B | \vec{p}) = P(\vec{n}_C | \vec{p})$, denoting $f(\vec{p}) = P(\vec{n}_B | \vec{p})$ and using that in our example p is one-dimensional, we have that (1) equals

$$\int_p f(p)^2 dp$$

and (2) equals

$$\left(\int_p f(p) dp \right)^2 .$$

As f is a function whose mass is concentrated on a small fraction of its support, (1) is larger than (2). Or, speaking less mathematically, modeling the two subtypes separately instead of jointly, gives the prior probability a larger influence which leads to $\vec{p}$ not well supported by the observed nucleotide frequencies becoming more probable.

References

1. Unterthiner T, Schultz AK, Bulla J, Morgenstern B, Stanke M, Bulla I: **Detection of viral sequence fragments of HIV-1 subfamilies yet unknown**. *BMC Bioinformatics* 2011, **12**:93.
